# How Do Incentives Lead to Deception in Advisor–Client Interactions? Explicit and Implicit Strategies of Self-Interested Deception

**DOI:** 10.3389/fpsyg.2012.00527

**Published:** 2012-12-04

**Authors:** Barbara Mackinger, Eva Jonas

**Affiliations:** ^1^Department of Psychology, Paris Lodron University SalzburgSalzburg, Austria

**Keywords:** strategic behavior, deception, self-interest, incentive, advice-giving, motivated information processing, principal-agent theory

## Abstract

When confronted with important questions we like to rely on the advice of experts. However, uncertainty can occur regarding advisors’ motivation to pursue self-interest and deceive the client. This can especially occur when the advisor has the possibility to receive an incentive by recommending a certain alternative. We investigated how the possibility to pursue self-interest led to explicit strategic behavior (bias in recommendation and transfer of information) and to implicit strategic behavior (bias in information processing: evaluation and memory). In Study 1 explicit strategic behavior could be identified: self-interested advisors recommended more often the self-serving alternative and transferred more self-interested biased information to their client compared to the advisor without specific interest. Also deception through implicit strategic behavior was identified: self-interested advisors biased the evaluation of information less in favor of the client compared to the control group. Self-interested advisors also remembered conflicting information regarding their self-interest worse compared to advisors without self-interest. In Study 2 beside self-interest we assessed accountability which interacted with self-interest and increased the bias: when accountability was high advisor’s self-interest led to higher explicit strategic behavior (less transfer of conflicting information), and to higher implicit strategic behavior (devaluated and remembered less conflicting information). Both studies identified implicit strategic behavior as mediator which can explain the relation between self-interest and explicit strategic behavior. Results of both studies suggest that self-interested advisors use explicit and implicit strategic behavior to receive an incentive. Thus, advisors do not only consciously inform their clients “self-interested,” but they are influenced unconsciously by biased information processing – a tendency which even increased with high accountability.

“If you want people to perform better, you reward them, right? Bonuses, commissions, their own reality show. Incentivize them.”

(Daniel Pink^1^)

This quote of a well known American career analyst explains one common strategy of motivating employes in the business world. It follows a simple analogy: sell or produce X for the company and you will get Y as a reward. Especially, at a time where companies are in trouble to survive on the market, they are challenged to perform well. Taking the competitive nature of the market into account, it is comprehensible that companies use incentives as an instrument to motivate employes. Incentives are assumed to encourage employes in accomplishing a stated goal, or even in following a goal that they would normally have no other reason for pursuing. Incentives should help by guiding self-interested behavior and adjusting the employes’ interests to the company’s interests.

Indeed, there are many examples showing that using incentives often work out well for companies. One company, which has been working successfully with incentives, is Tupperware. Their marketing principle of using Tupperware-parties to sell their products is very effective. These parties take place in the private atmosphere of someone’s home. The host provides the location, snacks, and refreshments, and invites the guests. Well, how can that be that a private person starts a marketing event for a company he or she does not work for? Back in the 1950s, bringing women together and discussing housekeeping and kitchen secrets was nothing new, but receiving additional incentives for hosting the event and for providing people who buy a lot, was an innovation. Today, the promise of incentives especially in form of turnover-dependent commission is still assumed to have a direct impact on peoples’ behavior. The host invites a lot of people, creates a nice surrounding atmosphere with sandwiches and drinks because he or she assumes that this behavior will pay off in the end.

However, the question of interest is whether incentives really just motivate employes or hosts of Tupperware-parties to behave in vein with the company’s strategic interest, or does it lead to more wide reaching consequences? For the company it is somehow desirable that the host behaves strategically in order to pursue self-interest to earn a lot of money. This type of strategic behavior is in line with the intention to sell a lot. Therefore, the company directs the hosts’/employes’ behavior by incentives which are adapted to its own interests. Thus, this type of strategic behavior might also lead to deceive the other party consciously: for example the host is recommending especially expensive products to the customers because he or she receives a turnover-dependent commission. For this reason, he or she may communicate only the advantages of the product and withhold the disadvantages (e.g., Buller and Burgoon, 1994, 1996; Steinel and De Dreu, 2004). This behavior is henceforth called *explicit strategic behavior*. Explained more in detail, people showing strategic behavior can have the explicit goal to deceive and therefore alter the customer’s behavior or opinion in order to receive an incentive.

However, we speculate that the promise of incentives could also entail unconscious risks. In order to gain reward, people might behave more implicitly strategic which supports the deception of the other party. Past research already showed that deception is also accompanied by more automatic and less conscious actions, such as smiling longer and nodding when deceiving our counterpart in face-to-face interaction (e.g., Buller and Burgoon, 1994; Burgoon et al., 1996). However, this is not an explicit strategy to deceive the other party. People simply seem to implicitly engage in this deception process. In our opinion, deception can also already start before the interaction with a counterpart, for example through biased information processing. This occurs when the host evaluates and remembers expensive products biased in favor of one’s possibility to receive more reward. We call the biased information processing henceforth *implicit strategic behavior*, which in contrast to explicit strategic behavior is not clearly linked to deception, because it is not used to alter the client’s behavior or thinking in order to increase their own incentives. However, we think that it is triggered by the wish to receive a high incentive and we therefore investigate it as an implicit strategic behavior of deceiving the other party.

As is generally known, the promise of incentives is not only common practice in occurrences like Tupperware-parties, where peer-advice is given. Incentives are also widespread in professional consultancy where advisors have specialized knowledge, which is demanded by clients to improve their decision (e.g., financial advisors, physicians, and personnel advisors). Clients are in a clear disadvantage because of their lack of knowledge and advisors can use these scopes to deceive the client in self-interested manner. In the current paper we investigate if the promise of incentives motivates advisors to make consciously use of their advantage and behave explicitly strategic by deceiving the other party. However, we assume advisors are also influenced by the incentive more implicitly, which should display in biased information processing. This is especially risky and therefore highly relevant for advisors who prepare information for their clients. So far, past research did not investigate this type of strategic behavior which might support the explicit strategic behavior to deceive a counterpart. We test our assumptions in two studies. Whereas our second study additionally examines to what extent incentivized advisors behave strategically when they feel highly accountable for their clients.

## Explicit Strategic Behavior

Strategic behavior is primarily used to create a false belief in order to deceive the other party. One theoretical background of deception is the Interpersonal Deception Theory (IDT, Buller and Burgoon, 1994, 1996), which focuses primarily on the face-to-face interaction and the dynamic of deceptive interaction between sender and receiver (verbal and non-verbal). Buller and Burgoon (1994) describe observable strategic behavior during the deception (e.g., ambiguous, vague, and intentional messages), which is used by the senders to alter the communication in order to achieve their goal.

An alternative description of deception and strategic behavior is proposed by the economic principle-agent theory (PAT; Ross, 1973), which focuses less on communication and more on strategic actions within working relationships. According to this theory, strategic behavior is likely to occur when one party (principal) delegates work to a more knowledgeable party (agent) and the two parties have different interests. In other words, the client is under uncertainty when asking a knowledgeable advisor for support to find the best solution for a problem, if the advisor really acts in client’s best interest or in their own interest. Advisors, in the role of an expert, have wide-ranging possibilities to use scopes to behave strategically and to pursue their self-interest. The client lacks the knowledge to fully evaluate the quality of the advisor’s recommendation. Therefore, the advisor tends to provide recommendations which support their own self-interest rather than the client’s interest.

Deception and the different levels of knowledge between two parties were already found as crucial in bargaining experiments especially when stakes were high (Boles et al., 2000): In this situation participants in the role of the proposer behaved strategically by offering less and providing a worse bargain to their unknown counterpart (who did not know the size of the pie) compared to a more knowledgeable counterpart. This means proposers pursued self-interest, when there was a low possibility to be detected by the counterpart and only when the stakes were high (Boles et al., 2000).

We would now like to turn to advice-giving situations, where likewise many opportunities exist to deceive the client. This should be illustrated with the help of an example of a personnel advisor: Companies hire a personnel advisor when needing support for a specific job placement and typically pay him or her with an agency fee after successful stuffing. This procedure, as typically used by companies, leads to an incentive for the advisor to find a suitable candidate as soon as possible in order to fulfill the contract and to receive the incentive (agency fee). In other words, a fast fulfillment of the contract enhances the chances of the advisor to soon be available for a new contract and a new possibility to earn money. Thus, for the company and the possible job applicant the risk exists that the advisor is rather interested in a fast than in the best job placement (goal conflict). Because of advisor’s information advance (e.g., job market, job duties, salary, and education), he or she can make use of existing scopes and deceive by behaving explicitly strategic. This information asymmetry is existing in many advisor–client interactions and similar behavior by the advisors can be assumed, such as physicians or financial advisors.

According to the PAT (Ross, 1973; for overview Eisenhardt, 1989), in such situations strategic behavior occurs in different forms, such as “Moral hazard,” withholding actions or information (“hidden action,” “hidden information”), and also in terms of “Hold up” (“hidden intention”), such as concealing the own goals and intentions. Similarly, IDT (Buller and Burgoon, 1994, 1996) list (explicit) strategic behaviors regarding the regulation of the content of the spoken information (“information management”). Deceivers, for instance, were found to behave strategically and use higher rates of irrelevant and vague information (Buller and Burgoon, 1994).

Indeed, further research shows that participants behave strategically by managing and controlling information. For example, Steinel and De Dreu (2004) found that participants of an information provision game behaved strategically. Participants were in the position to guide their counterpart which had opposing interests (losing points when the counterpart gain points) through passing accurate or inaccurate information. The aim of the participants was to reach as many points as possible, since these determined the amount of lottery tickets they would receive in the end. Results demonstrated that participants behaved explicitly strategic by withholding more accurate information and passing more inaccurate one. Additionally the authors could identify that strategic behavior was mainly driven by greed within this interdependent relationship with opposing interests (Steinel and De Dreu, 2004).

Looking back at the case of the personnel advisors this means that the possibility to receive an incentive for recommending a specific alternative may frequently lead to explicit strategic behavior: the advisor may offer only jobs to applicants which allows a quick recruitment process with low effort (PAT: “hidden action”). To achieve this, the advisor may strategically transfer information to the client to accelerate the process of convincing the client to accept the easily available job instead of prolonging the search for the best job alternative. This can occur by solely presenting those aspects of the easily available job which are in line with the applicant’s demands (good labor-market situation, career opportunities) or by withdrawing the conflicting information (salary, job characteristics) (PAT: “hidden information” and Steinel and De Dreu, 2004). Based on this assumption we assume the following hypothesis regarding the explicit strategic behavior:

Hypothesis 1: We assume that the self-interested advisors recommend the easily available job more often compared to the advisor without specific interest.

Hypothesis 2: We suppose that self-interested advisors enhance supporting information and devalue conflicting information regarding their self-interest whereas advisors without specific interest do not make this difference.

Well, if these predictions become true they are a large problem for advice-taking situations, where clients often have to rely on the knowledge of an advisor as expert. However, past deception research has only tested the conscious modification of information (PAT: Ross, 1973; IDT: Buller and Burgoon, 1996). Heretofore, it has failed to incorporate the more implicit biasing of the information processing within the deception. But we assume that the motivation to gain reward might influence the advisor even more implicit.

## Motivated Directional Goal and Implicit Strategic Behavior

First, before we fully understand implicit strategic behavior, we need to describe the process of advice-giving and to differentiate the phase of explicit advice-giving, and the preceding phase of preparing the recommendation (Jungermann, 1999). Within the latter one the more implicit information processing has to be taken into account. In this phase the advisors search, evaluate, and process information. At the latest since Kunda (1990) we know that there are motives, such as receiving an incentive, that influence our reasoning. This means people’s goals, wishes, fears, and desires lead people to engage in biased information processing and direct our thinking and convictions (Kruglanski, 1989; Kunda, 1990; Dunning, 1999; Kruglanski et al., 2012). Kruglanski et al. (2012) describes a directional motivation like a psychological force, similar to a physical force, which is determined by a specific desired goal. People perceive the world through their “motivated colored” glasses – perceiver’s goals are crucial for reconciling incoming information.

Past research provided evidence that such directional goals are also important for predicting advisors’ behavior in advice-giving processes (Jonas et al., 2005). This findings indicated that advisors, who had to justify their recommendation and therefore had an incentive to appear in a positive light in front of the client (impression motivation), biased their information search in favor of their recommendation and passed on more information supporting the recommendation (Jonas et al., 2005). In contrast, advisors without directional goals were found to act accuracy motivated and were normally directed by finding the best solution for their clients (Jonas and Frey, 2003). Therefore, only in order to reach the directional goal, making a good impression, advisors behaved implicitly strategic by searching information which primarily supported their recommendation.

Implicit strategic behavior can be further illustrated through the previously introduced example of the personnel advisor, who has the directional goal of fast contract fulfilling in order to receive the incentive quickly. An advisor who is preparing a recommendation for a job applicant, always has in mind which jobs are easily available at the moment and can be staffed quickly, allowing the advisor to earn more money. In this scenario earning money can be seen as a main motivation. To reach the directed goal of earning money, the advisor might behave implicitly strategic when evaluating job information. He or she might evaluate the information of an easily available job less critical than that of a difficult available job: the advisor may enhance job relevant information which is in line with his or her goal. Similarly, the advisor might also remember information more easily which supports his or her goal compared to conflicting information. This biased information processing might also implicitly influence the recommendation that is given.

Going beyond to the assumption of PAT (Ross, 1973) people’s motivation to reach a goal do not only lead to an actor’s obvious and conscious behavior (e.g., advisors strategic behavior – explicitly hiding information). Past research in motivated reasoning suggests that people engage in biased information processes because they find it more plausible to process information in line with their own beliefs and expectancies (e.g., McDonald and Hirt, 1997) and to remember desired aspects (e.g., Sanitioso et al., 1990). These findings indicated enhanced accessibility of knowledge structures, which are in line with the desired goal, so that, the personnel advisor might be caught in biased information processing, when trying to fulfill the contract quickly in order to receive the incentive. Even when the information processing is not directly and consciously used to receive an incentive, the personnel advisor will evaluate and maybe even remember information in favor of their self-interest. We propose that this phenomenon can be seen as an implicit and unconscious process – or in other words as implicit strategic behavior to get the promised incentive.

Interestingly, receiving incentives has already led to different assumptions regarding information processing and was discussed controversially in past research. On one hand, there is evidence that incentives lead to higher accuracy motivation and that people put more effort in information processing when receiving an incentive (Stone and Ziebart, 1995). On the other hand, incentives can also lead to a higher confirmation bias (Jonas et al., 2008): Participants who were promised an incentive for finding the correct answer showed a preference in searching for supportive rather than conflicting information regarding their preliminary decision. Moreover, they also remembered conflicting information worse. This research showed that incentivized participants were more biased in their information processing than participants without incentives. This poses the question of whether information processing is also biased in order to receive incentives. We assume that the incentives influence the advisor’s thinking and convictions in a similar manner as a directional goal. The personnel advisor might bias information processing in favor of their self-interest, or the job alternative which is associated with the incentives.

Independent from incentives, other research showed that self-interest had an influence on the information processing, such as the evaluation and memory of self-interested information (Kunda, 1990). In a study of Ditto et al. (1998) participants were tested by means of a clinical test, which had either positive or negative consequences for the participants’ health. Participants had to evaluate the test and their test results. The results showed that they were rather dismissive when they were confronted with negative health consequences, compared to participants who faced a result with a positive health consequence. Additionally, based on the research of Kunda and colleagues (e.g., Kunda, 1987; Kunda and Sanitioso, 1989; Sanitioso et al., 1990) we know that our self-interest also influences our memory search. For instance, participants who were persuaded that introversion (or extraversion) is more desirable for academic success described themselves as more introverted (or extroverted). Furthermore, they were able to report more and faster introverted (or extroverted) behavior pattern than the participants who had been convinced of the opposite (Sanitioso et al., 1990).

Similar results were found in the field of the persuasion research (Petty and Cacioppo, 1990) where people’s different involvements and benefits (vested interest) from actions in society led to different evaluations of information (outcome-relevant involvement, Johnson and Eagly, 1989). Four experimental studies by Darke and Chaiken (2005) showed that self-interest influences the direction of attitudes and the persuasive impact of arguments: participants, who had to pay the costs and did not receive any benefits, devalued the new policy (tuition fees) by processing the information of the arguments in a biased way. In similar vein the research in motivated skepticism of political beliefs found evidence that people used biased information processing as means of finding consistency with their own favored view (Taber and Lodge, 2006). Participants who felt strongly about an issue – even when encouraged to be objective – evaluated supportive arguments more favorably than conflicting arguments. This research indicates that people in social interactions such as discussing new policies are biased through their self-interest. They want to bolster their view and find consistency for the own favored view.

However, this research does not state how people are influenced by their self-interest when processing information for another person and preparing an advice. In the present study, we want to investigate how self-interest in the form of receiving incentives biases people’s information processing and implicitly influence the deception in advice-giving. Furthermore, advisors may put more focus and effort in understanding the match between an applicant and an easily available job compared to other job alternatives, which may display in a biased memory. We suppose this also in our following hypothesis.

Hypothesis 3: We propose that self-interested advisors enhance the relevance of supporting information and devalue the conflicting information regarding their self-interest whereas advisors without specific interest do not make this difference.

Hypothesis 4: We suppose that self-interested advisors remember conflicting information regarding their self-interest worse, than advisors without specific interest.

Finally, it remains the question how implicit and explicit strategic behaviors are connected and how advisor’s implicit biases shapes self-serving deceptive behavior. A recent work Shalvi et al. (2011) assessed deception in participants who were asked to privately roll a die under a paper cup three times versus only once. Afterward they reported the outcome of the first roll and gain money as a function of their reports (1 = $1, 2 = $2, etc.). Results suggested that the degree of lying was significant higher in the condition with three times compared to once rolling the die because of the higher extent of self-justification by referring to the highest outcome of the three rolls. In sum, self-interest led people to view objective information in a biased way which supported their self-justification and enabled them to lie. Specifically, the authors assume that people balance their desire to profit from the lie with a desire to maintain their self-concept as honest individuals. Similar in our study, self-interest bias in information processing (implicit strategic behavior) enables the advisor to justify the later self-interested explicit strategic behavior. Therefore we propose that implicit strategic behavior should help to describe the process of explicit strategic behavior.

Hypothesis 5: We assume that the connection between self-interest and explicit strategic behavior (transfer of information) is mediated to some extent by implicit strategic behavior (evaluation of information).

## Present Research

In order to assess the outlined hypotheses, in our first study we asked participants to put themselves in the role of either self-interested personnel advisor or a personnel advisor without self-interest. The scenario was similar as already introduced in the beginning. However, in our second study we want to enhance advisor’s responsibility and accountability for their recommendation. Therefore, besides self-interest we investigated advisor’s accountability and how this influenced explicit and implicit strategic behavior.

## Study 1

### Method

#### Participants and design

Participants were 67 students (54 female, 13 male) at a public university of Austria (University of Salzburg). Psychology students could volunteer in order to receive credits for participation. They participated individually. The study was a two factorial design with two conditions 2(self-interest: yes vs. no) × 2(type of information: supporting vs. conflicting).

#### Scenario and task

After participants consented to being in the study, they were placed in a quiet area. The questionnaire started with the description of the personnel advisor’s scenario. Part of the scenario was a fictitious client. He was described as a young male high-school graduate that is interested in the field of engineering. The advisor got informed that different tests could already confirm the appropriateness of this client in this field and about some important specific details which should be taken into account for the recommendation (e.g., salary as important criterion, above the average in logical reasoning, loves challenge in logical thinking, below average in the ability to work, and cooperate in teams).

Participants assumed either the role of a freelancer personnel advisor with the possibility to earn an incentive when pursuing self-interest (*self-interested advisor*), or a personnel advisor of an institution for professional training (*advisor without specific interest*). Only the self-interested advisor is also under contract of a company, which commissioned the advisor to find an appropriate candidate for the job of a *product engineer*. Therefore the advisor could pursue self-interest and receive an incentive by recommending this job to the client. Participants also pursued real self-interest – they only participated in lottery to win one of ten 20€-Amazon-voucher if they recommended the product engineer. In the other condition the advisor had no additional interest to fulfill a contract with a company in order to receive an incentive and also participants themselves took part in the lottery independently of their recommendation.

The assignment for the career consultant was then to read information about three vocational trainings for the client: machinery engineer, mechatronic engineer, or product engineer. For preparing the recommendation, the participants had to evaluate the information. Further they expressed their intention of transferring information to the client and finally recommended one job. In the end, the participants answered questions regarding their perceived self-interest and took part in a quiz regarding the job information.

### Material

#### Job information – conflicting and supporting information

Information covered six categories for the three different job possibilities (see Table A1 in Appendix) and there are clear opposed interests between the client and the self-interested advisor. On the one hand, the mechatronic engineer’s job description fitted best with the client’s demands – it covered most of his needs and wishes (high salary, logic reasoning is important, and working in teams is not mentioned as key competence). On the other hand, the product engineer is the best option for the advisor to meet his/her self-interest. The information about the machinery engineer were similar attractive to the product engineer – both had three pieces of information which covered the wishes of the client and three which were in conflict.

For further analysis, the pieces of information are used as either supportive or conflicting with advisor’s self-interest. Conflicting information are all information which weaken the realization of the advisor’s self-interested goal (negative arguments for the product engineer, positive arguments for the mechtronic, and maschinery engineer). In contrast, supportive information contains all arguments which can bolster the self-interested recommendation of the product engineer (positive arguments for the product engineer, negative arguments for the mechatronic, and maschinery engineer). We want to refer to Table A1 in Appendix where we signed supportive arguments for the advisor with plus and conflicting information of information with minus.

#### Explicit strategy

##### Transfer of information

Participants marked on a 10 cm line how likely they would hand this information to their client. For further analysis we divided the scale in the transfer of the supporting (six items, Cronbach’s α = 0.72) and conflicting information (11 items, Cronbach’s α = 0.90).

##### Recommendation

Additionally, after reading all information the participants had to decide for one specific job – product engineer, mechatronic engineer, and machinery engineer – which she or he would recommend to the client.

#### Implicit strategy

##### Evaluation of information

The participants in the role of the advisor had to decide how relevant the job information is. They marked their evaluation on a 10 cm line which reached from not relevant to very relevant. For our further analysis we used the evaluated relevance of the supporting (six items, Cronbach’s α = 0.83) and the conflicting information (11 items, Cronbach’s α = 0.85) regarding the advisor’s self-interest.

##### Memorizing information

Subsequently the participants were requested to answer six multiple choice quiz questions regarding information of the three different job alternatives to measure how much information was memorized. Actually, we used only a single-item measure for further analysis; however it is the most conflicting information regarding the advisor’s self-interest (salary of the mechatronic engineer).

#### Manipulation check

##### Perceived self-interest

Additionally, the perceived own self-interested behavior was measured with the scales *hidden intention* (e.g., “Situations where the client’s and my interests were in conflict, I oriented primarily on my interests.” Five items, Cronbach’s α = 0.92), *hidden information* (e.g., “Some important information were not communicated to the client.” Four items, Cronbach’s α = 0.83) and *hidden action* (e.g., “Some actions were more in my interest than in the interest of the client.” Two items, *r*(66) = 0.65, *p* < 0.01). Participants answered by responding to the questions on a five-point Likert-scale (1 = strongly disagree to 5 = strongly agree). For further analysis we combined these three scales and measured self-interested behavior in general (11 items, Cronbach’s α = 0.95).

## Results

### Manipulation check

For checking the influence of the manipulation we used the scale general perceived self-interest. Analysis regarding the influence of the manipulated self-interest revealed a main effect, *F*(1,67) = 18.17, *p* < 0.001. The freelancer advisor perceived him/herself as significant more self-interested compared to the personnel advisor of an institution for professional training without any specific self-interest (*M*s = 2.72 vs. 1.84, *SD*s = 1.09 vs. 0.47). Thus, this result suggests that the intended factors were manipulated successfully.

### Explicit strategies

Based on the assumption that the advisor is influenced by the promise to receive an incentive in a self-interested manner, we expected an explicit strategic behavior in the explicit recommendation to the client and in the transfer of information. The self-interested transfer of information should be characterized by withholding conflicting information regarding the self-interest and pushing forward supporting information.

#### Advice-giving – Hypothesis 1

In line with our first hypothesis a Chi-squared test on advice-giving strategy displayed that participants in the role of self-interested freelancer advisor recommended significant more often the less appropriate option “product engineer” to their client than participants without self-interest, χ^2^(1, *N* = 67) = 8.49, *p* = 0.04 (self-interest: 10 product engineer, 24 no product engineer; without self-interest: 1 product engineer, 32 no product engineer). The result supported our assumption that participants who had a personal self-interest are influenced in advice-giving and recommended significantly more often the product engineer, which is the self-interested alternative for the advisor. Additionally, Chi-squared analysis with all three job alternatives showed that advisors with no specific interest recommended more often the optimal job to their client (“mechatronic engineer”) than participants with self-interest, χ^2^(1, *N* = 67) = 8.61, *p* = 0.014 (without self-interest: mechatronic 30, product 1, machinery 2 vs. self-interest: mechatronic 23, product 10, machinery 1). These results support our hypothesis that self-interested advisors recommend the self-interested alternative of the product engineer more often compared to advisors without specific interest. Additionally advisors without specific interest recommended the optimal job more often than those with self-interest.

#### Transfer of information – Hypothesis 2

**Figure 1:**
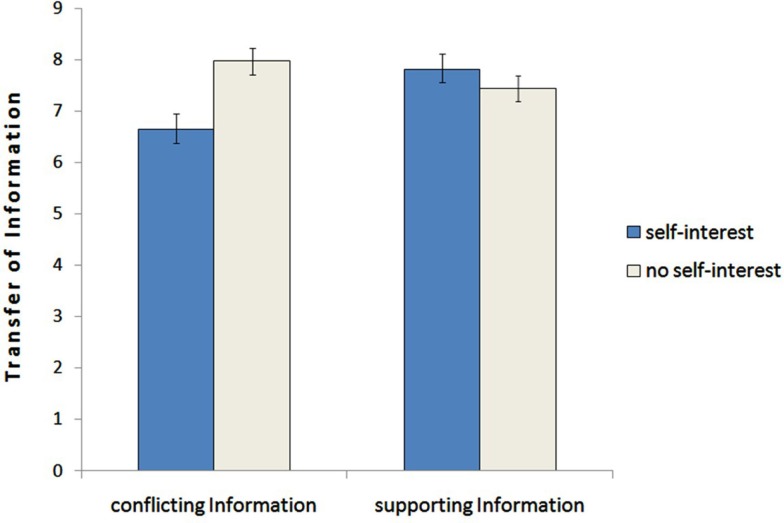
**Mean differences representing the transfer of information regarding supportive and conflicting information for advisors with self-interest and without self-interest**. The error bars represent SEM (study1).

To test this hypothesis we ran a 2 (self-interest: yes vs. no) × 2 (information: supporting vs. conflicting) analysis of variance with repeated measures on the last factor. This analysis revealed no main effect for the information, *F*(1,65) = 1.91, *p* = 0.17, $\eta_{p}^{2}=0.029$ (supporting: *M* = 7.62, *SD* = 1.48 vs. conflicting: *M* = 7.30, *SD* = 1.89). However, the analysis displayed a significant interaction effect between job information and self-interest, *F*(1,65) = 13.50, *p* < 0.001, $\eta_{p}^{2}=0.17.$ Subsequent *post hoc* analysis indicated that results are in line with our predictions: Participants in the in the role of the self-interested advisor transferred less conflicting information (*M* = 6.64, *SD* = 2.23) than the advisor without specific interest (*M* = 7.97, *SD* = 1.16), *F*(1,65) = 9.20, *p* = 0.003. Additionally, self-interested advisors passed significant more information which supported their self-interest to the client than information which conflicted their self-interest (*M* = 7.81, *SD* = 1.54 vs. *M* = 6.65, *SD* = 2.23), *F*(1,65) = 12.98, *p* = 0.001. There was a tendency that advisors without specific interest transferred even less supporting than conflicting information (*M* = 7.44, *SD* = 1.41 vs. *M* = 7.97, *SD* = 1.55), *F*(1,65) = 1.04, *p* = 0.112. Regarding the supporting information there was no significant difference between advisors with self-interest and without specific self-interest (*M* = 7.81, *SD* = 1.54 vs. *M* = 7.44, *SD* = 1.41) *F*(1,65) = 1.04, *p* = 0.311. This indicates that advisors with self-interest primarily withhold conflicting information and did not transfer more supporting, whereas advisors without specific interest transferred information more balanced – with a contrary tendency to transfer more conflicting than supporting information. Results are displayed in Figure 1.

### Implicit strategies

Well, besides explicitly strategic advice-giving we predict that advisor’s information processing is also influenced by incentives. The promise of incentives leads to self-interested bias in the evaluation of information and in memorizing the information (supporting vs. conflicting information regarding advisor’s self-interest).

#### Evaluation of information – Hypothesis 3

**Figure 2:**
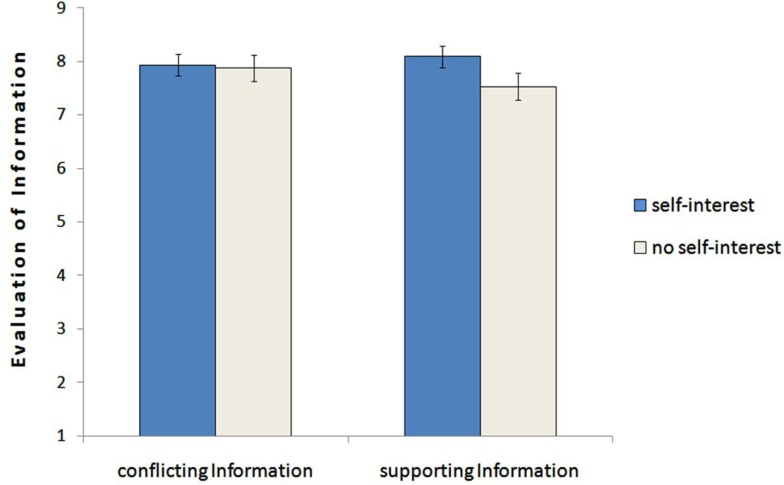
**Mean differences representing the evaluation of information regarding supportive and conflicting information for advisors with self-interest and without self-interest**. The error bars represent SEM (study 1).

To examine the effect of the self-interest on the evaluation of the information, we ran a 2 (self-interest: yes vs. no) × 2 (information: conflicting vs. supporting) analysis of variance with repeated measures on the last factor. This analysis revealed no main effect for the information, *F*(1,65) = 0.67, *p* = 0.42, $\eta_{p}^{2}=0.10$ (conflicting: *M* = 7.90, *SD* = 1.16 vs. supporting: *M* = 7.82, *SD* = 1.44). However, it showed a significant interaction effect between job information and self-interest, *F*(1,65) = 4.97, *p* = 0.029, $\eta_{p}^{2}=0.07.$ *Post hoc* analysis verified the pattern that there was a tendency for supporting information to be higher evaluated by the self-interested advisor than by the advisor without special interest (*M* = 8.09, *SD* = 1.41 vs. *M* = 7.53, *SD* = 1.43), *F*(1,65) = 2.57, *p* = 0.114. In contrast, the conflicting information was evaluated similarly in its relevance by the self-interested advisor and the advisor without interest (*M* = 7.93, *SD* = 1.30 vs. *M* = 7.87, *SD* = 1.02), *F*(1,65) = 0.04, *p* = 0.835. However, advisors without specific self-interest devaluated supporting information significant compared to conflicting information (*M* = 7.53, *SD* = 1.43, vs. *M* = 7.87, *SD* = 1.02), *F*(1,65) = 4.56, *p* = 0.036; advisors with self-interest did not evaluate supporting and conflicting information significantly different (*M* = 8.09, *SD* = 1.41, vs. *M* = 7.93, S*D* = 1.30), *F*(1,65) = 1.01, *p* = 0.318. The hypothesis gets support by the significant interaction between self-interest and type of information, whereas the interaction is mainly driven by the enhanced evaluation of the conflicting information compared to the supporting information within advisors without specific interest. It seems that this distinction regarding the evaluation of the information disappears when pursuing self-interest. Results are displayed in Figure 2.

#### Memorized information – Hypothesis 4

Further, we tested the influence of self-interest on the memorized information. The results indicated that self-interested participants remembered significantly worse that the mechatronic engineer had the best possibilities to receive a good salary (conflicting information), *t*(65) = 2.00, *p* = 0.05 (self-interest *M* = 3.79, *SD* = 0.59; without self-interest *M* = 4.00, *SD* = 0.00). Really remarkable was that each participant without specific could remember the correct answer.

**Figure 3:**
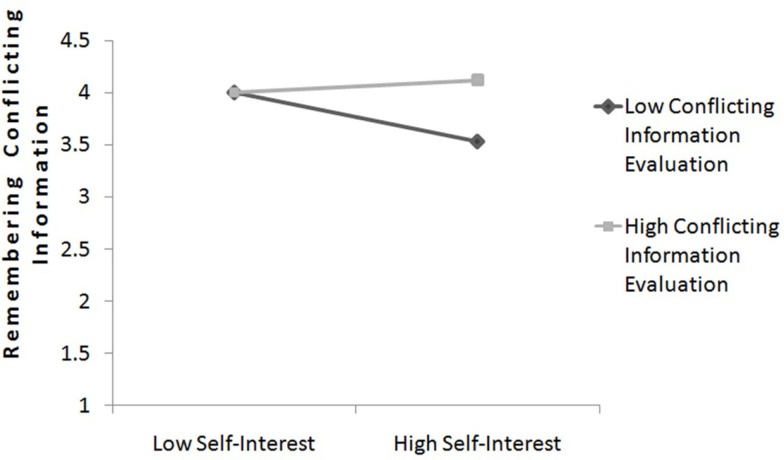
**The relationship between self-interest and remembering conflicting information as a function of the evaluation of conflicting information (study 1)**.

For further exploratory analysis of our data we used the pursued self-interest^2^ together with the devaluation of conflicting information to predict biased memorized information (salary of the mechatronic engineer). We conducted a hierarchical regression analysis in which memorized conflicting information was predicted by main-effect terms (evaluation of the conflicting information and self-interest) and the interaction term simultaneously. Following Aiken and West (1991), the variables evaluation of conflicting information and self-interest were centered (i.e., by subtracting the mean from each score), and the interaction term was based on these centered scores. The interaction between evaluation of the conflicting information and self-interest was significant, *b* = 0.32, SE = 0.10 *t*(63) = 3.18, *p* = 0.002. Simple slope analysis was conducted to further analyze this interaction (Aiken and West, 1991). When the relevance of the conflicting information was high (1 SD above the mean), self-interest was not significantly related to memorized information, *b* = 0.22, SE = 0.16, *t*(63) = 1.39, *p* = 0.170, which means among participants who evaluated conflicting information high self-interest had no specific influence on the memorized knowledge. However, when the relevance of conflicting information was evaluated low (1 SD below the mean; *b* = −0.43, SE = 0.15 *t*(63) = −2.91, *p* = 0.005), self-interest was associated with less memorized information. The slopes are plotted in Figure 3.

#### Mediation – Hypothesis 5

We assumed that the connection between self-interest and explicit strategic behavior can be explained to some extent by implicit strategic behavior. Therefore we conducted a mediation model with the implicit strategic behavior (evaluation of conflicting information) as a potential mediator, which should help to explain the relation between self-interest and explicit strategic behavior (transfer of conflicting information). The first regression analyses showed that self-interest was significantly associated with the potential mediator implicit strategic behavior, *b* = −0.31, SE = 0.12, *t*(67) = −2.59, *p* = 0.012. In the second step we tested whether implicit strategic behavior was significantly associated with the explicit strategic behavior – and indeed, implicit strategic behavior significantly predicted the explicit strategic behavior, *b* = 0.38, SE = 0.06, *t*(67) = 6.10, *p* < 0.001. In the final step we examined whether statistical control for the potential mediator reduced the predictive power of the relation between self-interest and explicit strategic behavior. Without the mediator the effect was significant, *b* = −0.80, SE = 0.08, *t*(67) = −10.68, *p* < 0.001, however, when controlling for the mediator the relationship was considerably reduced, *b* = −0.68, SE = 0.06, *t*(67) = −10.83, *p* < 0.001. Finally, in a bootstrap analysis implicit strategic behavior significantly carried the indirect effect, 95% CI = −0.24 to −0.02. Thus, evidence was found that the direct effect of self-interest on the explicit strategic behavior occurred partly through the implicit strategic behavior, which supports our Hypothesis 5.

## Discussion Study 1

Our results indicate that advisors with self-interest behaved explicitly strategic by recommending the self-interested alternative of the product engineer more often compared to advisors without specific interest. The self-interested advisor also transferred less conflicting than supporting information to the client, as well as self-interested advisors transferred less conflicting information compared to advisors without self-interest.

Self-interested advisors also behaved implicitly strategic. The evaluation of information led in advisors without specific interest to a significant enhanced evaluation of the conflicting information (supporting for the client, see Table A1 in Appendix) compared to the supporting information (conflicting for the client). This pattern displays the evaluation of information when having the best interest of the client in mind. The significant differentiation disappeared in advisors with self-interest. They did not take the perspective of the client and his needs and therefore, evaluated conflicting and supporting as similar important. Furthermore, we could confirm direct influence of self-interest on advisors’ biased memory. However, the investigation should be improved in Study 2, because in Study 1 we could only refer to one quiz question. Additionally, we could identify evaluation of conflicting information as moderator. Especially when the relevance of conflicting information was devalued self-interest had a significant negative influence on memorizing conflicting information.

With regard to the mediation analysis we found important evidence for the connection between implicit and explicit strategic behavior. Our results indicate that implicit strategic behavior can partly explain the relation between self-interest and explicit strategic behavior. This finding supports our assumptions that incentives have profound effects which influence people more implicitly and not only explicitly as assumed by the usual practice of incentives.

## Study 2

In study 1 our hypotheses received support from the experimental data which indicated that advisors with self-interest deceived the client by explicit and implicit strategic behavior. However, because of the hypothetical nature of the experiment participants of Study 1 could not get the impression that their advice would really help or harm a real client. Because of this lack of accountability the results of Study 1 could have been overestimated. Further clarification therefore is needed. In order to do this, we would like to more carefully look at the concept of accountability. Accountability is an expectation (implicit or explicit) that one may be called on to justify ones actions to others (Lerner and Tetlock, 1999). In practice, advisors are in this situation to justify their recommendation and action. But how does enhanced accountability influence advisor’s self-interested behavior?

One assumption could be that enhanced accountability leads to reduced self-interested behavior and consequently reduced explicit and implicit strategic behavior. Research findings can indicate that persons who are asked to justify their decisions are more likely to be interested in others outcomes. People with high endowment but having no accountability for group members contributed the same amount to a common system compared to those with few endowments. However, when they were accountable they made higher payments which helped in social dilemma situations (De Cremer and Van Dijk, 2009).

However, based on the review of Lerner and Tetlock (1999) we know it is especially necessary to take a closer look on the conditions of accountability. This review identified different conditions where accountability led to diverse outcomes in decision making and especially identified outcome vs. process accountability as crucial in this context (Lerner and Tetlock, 1999). Especially when people had to justify their outcome, such as their recommendation, they tended to increase their need of self-justification as well as biased information processing (e.g., Simonson and Staw, 1992). Contrarily accountability for decision processes led to more balanced evaluation when confronted with different alternatives. Consequently, advisors’ perceived accountability for their decision and expected need to justify this outcome should also lead to enhanced bias in information processing.

An additional closer look on the conditions for accountability in advice-giving situation is provided by research of Jonas et al. (2005). This study investigated the information search and transfer of highly accountable advisors (who assumed to meet the client and have to justify the recommendation) compared to advisors without accountability for their decision. This research found an enhanced confirmation bias for advisors’ binding recommendation when they were highly accountable for their decision but not in advisors without accountability. This effect could be explained by the directional goal of impression motivation. This means advisors wanted to appear in a positive light and therefore searched and also transferred primarily that information which was in line with their preliminary decision. This strategy helped them to provide evidence for their recommendation which supported their wish to present themselves in a positive way in front of their clients.

When referring to a self-interested advisor who has the directional goal to earn money we know already from Study 1 that they will evaluate and transfer information in a biased way in order to favor their self-interest. They also provide mainly information for their self-interested recommendation. However, how do advisors process information when they perceive themselves as both self-interested as well as highly accountable? We know from Study 1 that self-interested advisors who feel motivated by the goal to receive an incentive commit themselves already with the self-interested alternative before searching, evaluating and transferring information. In Study 1 this led to a bias in explicit and implicit strategic behavior. The perception of high accountability for their decision might increase the advisors’ wish to bolster their view. However, the salience of accountability might also counteract and reduce the self-interested bias in participants. Yet, given former research this latter alternative seems unlikely because being accountable *for an outcome*, such as a recommendation, has been shown to increase bias in information processing. Similarly, the *presence of a directional goal* (impression motivation) has also been shown to increase bias in information processing and information transfer. In Study 2 we investigate the influence of combining the presence of perceived accountability with self-interest on biased information processing and transfer. Therefore we tested the following hypotheses:

### Explicit strategic behavior

Hypothesis 6 – Transfer of information: We suppose especially among accountable participants, that self-interested advisors transfer less conflicting information compared to advisors without self-interest, this difference should be weaker within participants who are not accountable.

### Implicit strategic behavior

Hypothesis 7 – Evaluation of information: We assume among accountable participants, that self-interested advisors devalue conflicting information compared to advisors without self-interest, this difference should be weaker within participants who are not accountable.

Hypothesis 8 – Memory of information: Again we suppose especially among accountable participants, that self-interested advisors remember conflicting information less compared to advisors without self-interest, this difference should be weaker within participants who are not accountable.

### Moderated mediation

Hypothesis 9 – Transfer of information: We propose the indirect effect of self-interest on explicit strategic behavior (transfer of conflicting information) through implicit strategic behavior (evaluation of conflicting information) would be stronger under high than low accountability because accountability moderates the relation between self-interest and the mediator implicit strategic behavior.

## Method

### Participants and procedure

Participants were 53 students (36 female, 17 male) at a public university of Austria (University of Salzburg). The present study took place after a social psychology lecture. Psychology students could volunteer in order to receive credits for participation. The procedure in this experiment was similar to Study 1, with the following exception that we tried to manipulate accountability through the following sentence: “Please leave your e-mail address (on an extra sheet), so that the client can contact you for further questions.” The condition without accountability did not have this sentence in the questionnaire. Unfortunately our attempt to additionally manipulate accountability failed, *F*(3,53) = 0.36, *p* = 0.552. The survey took place in a huge lecturer hall where our manipulation was to weak. Although, the manipulation of self-interest was successful, *F*(3,53) = 3.16, *p* = 0.018, we use perceived self-interest and perceived accountability for further analysis. We discuss this decision later with our findings.

### Measures

#### Explicit strategic behavior

Again, we measured the intention to *transfer information* to the client, but used this time a five-point Likert-scale (unlikely to very likely). We used conflicting information (Cronbachs’α = 0.75) regarding self-interest for further analysis.

#### Implicit strategic behavior

For the *evaluation of the information* we applied a five-point Likert-scale (not relevant to very relevant). Conflicting information (Cronbachs’α = 0.67) regarding self-interest (see Table A1 in Appendix) is used for our further analysis. Further, we implemented a quiz to measure the *memorized information*, but we increased the amount of questions from 6 to 11. For further analysis we used only the conflicting information (six items, e.g., career opportunities for the mechatronic engineer, product engineer’s problems with the labor market) plus one question where participants had to remember the amount of salary of the mechatronic engineer. However, this question had no correct answer alternative – there was an optimistic (more than 30,000€ per year) vs. two rather pessimistic (not even 30,000€, at the best 30,000€ per year) and one neutral (approximately 30,000€ per year) biased alternative. For our conflicting information scale we added the optimistic alternative as correct answer.

#### Perceived self-interest

Similar to Study 1 we combined the three subscales of hidden intention, hidden information and hidden action and used one general scale of self-interested behavior for further analysis (nine items, Cronbachs’α = 0.91).

#### Perceived accountability

In the past research accountability was often manipulated through justification in front of a real audience. In our case we did not have real audience but some of the participants assumed further contact with the client per e-mail (attempt of manipulation). However, this typical accountability situation should be represented through our two questions which measures perceived accountability. (“How realistic was the situation to give advice to another person?” and “How accountable did you feel for your advice?” two items; *r* = 0.40, *p* < 0.01).

## Results

### Explicit strategic behavior

#### Transfer of Information – Hypothesis 6

We conducted a hierarchical regression analysis in which the transfer of conflicting information was conducted by perceived accountability and perceived self-interest (main-effect terms) and the interaction term simultaneously. Following Aiken and West (1991), the variables accountability and self-interest were centered (i.e., by subtracting the mean from each score), and the interaction term was based on these centered scores. The interaction between accountability and self-interest was significant, *b* = −0.14, SE = 0.06, *t*(49) = −2.25, *p* = 0.029, and as well a main effect for self-interest revealed significance, *b* = −0.34, SE = 0.08, *t*(49) = −4.25, *p* < 0.001. Simple slope analysis was conducted to further analyze the interaction (Aiken and West, 1991). When accountability was low (1 SD below the mean), self-interest was significantly negative related to the transfer of conflicting information, *b* = −0.20, *S*E = 0.08, *t*(49) = −2.37, *p* = 0.022. Therefore participants with low accountability were significantly influenced by their self-interest and passed on less conflicting information. However, when accountability was perceived high (1 SD above the mean; *b* = −0.49, SE = 0.12, *t*(49) = −4.08, *p* < 0.001), the relation between self-interest and less transfer of conflicting information even increased, which means an enhanced bias when accountability was high. However, the bias already existed when accountability was low, but high accountability increased the bias significantly.

**Figure 4:**
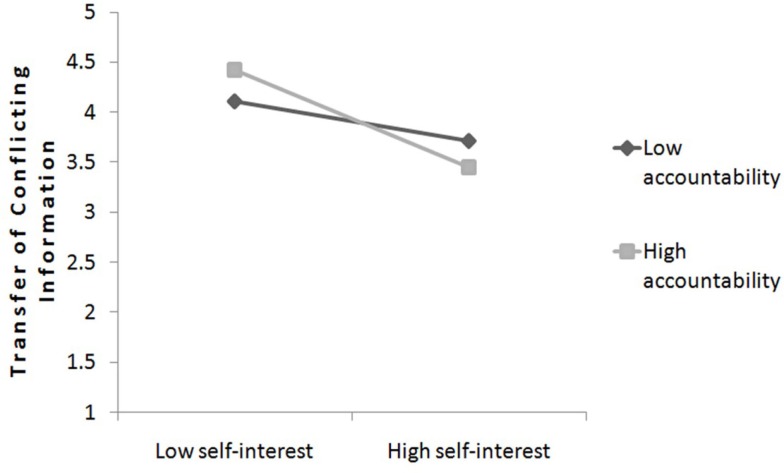
**The relationship between self-interest and transferring conflicting information as a function of advisor’s perceived accountability (study 2)**.

Additional data analysis showed, that this effect was similarly found regarding the general transfer of information (all information – conflicting and supportive), which indicates that among highly accountable advisors self-interest led to general withholding information [self-interest × accountability: *b* = −0.17, SE = 0.69, *t*(49) = −2.54, *p* = 0.015, 1 SD above: *b* = −0.60, SE = 0.13, *t*(49) = −4.66, *p* > 0.001, 1 SD below: *b* = −0.25, SE = 0.09, *t*(49) = −2.77, *p* > 0.001]. These results provided evidence that among advisors with high accountability, especially high self-interest led to withhold of conflicting information, which supports our Hypothesis 6. Moreover, our results indicate that self-interested advisors withhold general information and provide less information to their clients as advisors without self-interest. In other word self-interested advisors with high accountability do not distinguish between conflicting and supporting information and withhold information in general. The slopes are plotted in Figure 4.

### Implicit strategic behavior

#### Information evaluation – Hypothesis 7

**Figure 5:**
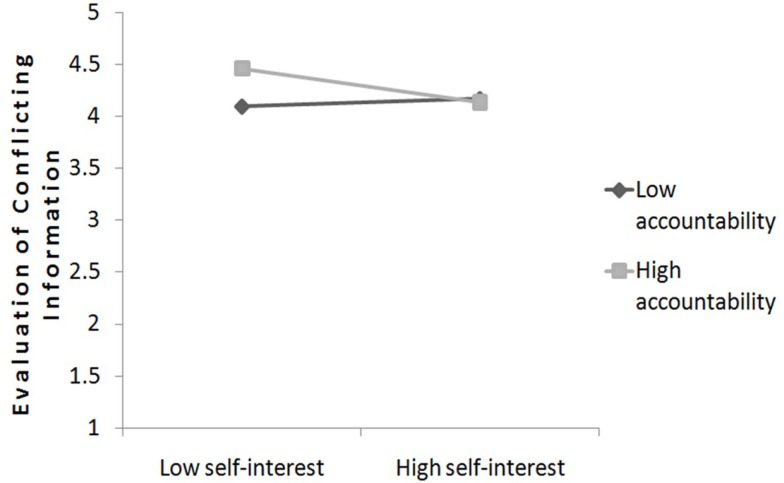
**The relationship between self-interest and evaluation of conflicting information as a function of advisor’s perceived accountability (study 2)**.

To test the perceived accountability as moderator between self-interest and the evaluation of conflicting information, we applied the same approach as already explained. The interaction between accountability and self-interest was marginally significant, *b* = −0.10, SE = 0.05, *t*(49) = −1.93, *p* = 0.060. Simple slope analysis was conducted to further analyze this interaction (Aiken and West, 1991). When accountability was low (1 SD below the mean), self-interest was not significantly related to the evaluation of conflicting information, *b* = 0.04, SE = 0.10, *t*(49) = 0.53, *p* = 0.596, in other words self-interest had no specific influence on the evaluation of conflicting information. However, when accountability was evaluated high [1 SD above the mean; *b* = −0.16, SE = 0.10, *t*(49) = −1.70, *p* = 0.097], self-interest was associated negatively with evaluated conflicting information. These results indicate that self-interested people under high accountability devaluate information compared to low self-interested participants, whereas participants with low accountability showed a similar level of devaluation regarding conflicting information. Therefore, these results do not suppose an enhanced bias compared to low accountability, however an enhanced bias between low and high self-interest among high accountability which supports the Hypothesis 7. The slopes are plotted in Figure 5.

#### Remembered information – Hypothesis 8

**Figure 6:**
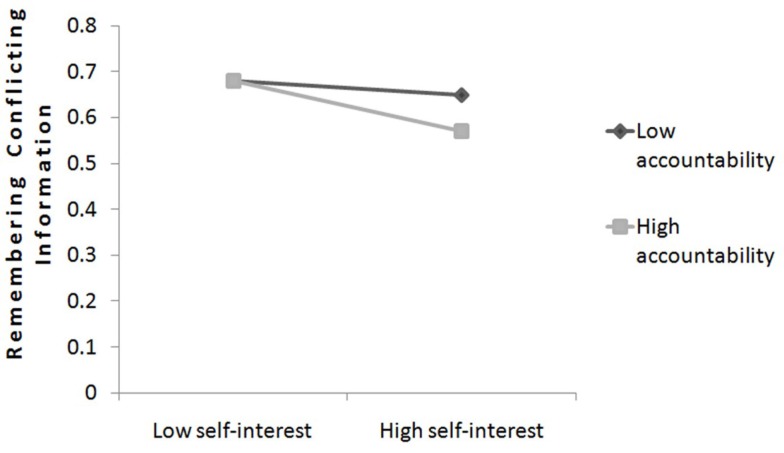
**The relationship between self-interest and remembering conflicting information as a function of advisor’s perceived accountability (study 2)**.

Accountability should be also tested as moderator between self-interest and the memorized conflicting information. We conducted a hierarchical regression analysis in which the memorized conflicting information was predicted by main-effect terms (perceived accountability and perceived self-interest) and the interaction term simultaneously. There was a significant main effect for self-interest, *b* = −0.04, SE = 0.01, *t*(49) = −2.52, *p* = 0.015 and the interaction between accountability and self-interest was marginally significant, *b* = −0.02, SE = 0.01, *t*(49) = −1.75, *p* = 0.086. Simple slope analysis was conducted to further analyze this interaction (Aiken and West, 1991). When accountability was low (1 SD below the mean), self-interest was not significantly related to memorized information, *b* = 0.02, SE = 0.02, *t*(49) = −1.09, *p* = 0.283, which imply when participants perceived themselves as less accountable self-interest had no specific influence on memory of conflicting information. However, when accountability was perceived high [1 SD above the mean; *b* = −0.06, SE = 0.02, *t*(49) = −2.64, *p* = 0.011], self-interest was associated significant negatively with memorized information. Therefore, among advisors with high accountability and high self-interest showed the worst memory regarding conflicting information. Accountability can be identified as marginal significant moderator which increases the self-interested bias in memorized conflicting information and therefore supports our Hypothesis 8. The slopes are plotted in Figure 6.

#### Moderated mediation – Hypothesis 9

We employed Preacher et al. (2007) (Model 2) bootstrapping procedure to test our moderated mediation hypothesis that the indirect effect of self-interest on explicit strategic behavior (transfer of conflicting information) through implicit strategic behavior (evaluation of conflicting information) would be stronger under high than low accountability because accountability moderates the relation between self-interest and implicit strategic behavior. As we already know the moderated regression analysis confirmed a marginal significant interaction between accountability and self-interest on implicit strategic behavior (see above Hypothesis 7). Using 1000 resample, analyses showed that implicit strategic behavior significantly mediated the effect of perceived self-interest on explicit strategic behavior under high accountability (90% CI: −0.25 to −0.01) but not under low accountability (90% CI: -0.04 to 0.11).

## Discussion Study 2

Our results indeed showed an interaction between self-interest and accountability indicating that high accountability enhanced the effect between self-interest and explicit (transfer of conflicting information) as well as implicit strategic behavior (evaluation and memory of conflicting information). More specific, we found that self-interested advisors increased their explicit strategic behavior by withholding information in general, but high accountability in advisors without self-interest led even to a reduced bias. This mean only the combination of high self-interest and high accountability led to increase in self-interested bias. This interaction was also found regarding implicit strategic behavior. Self-interested participants devaluated conflicting information only when they perceived themselves as highly accountable. High accountability without self-interest also led again to a reduced bias. Referring to the memory performance, self-interested advisors showed generally decreased performance regarding conflicting information. However, performance was especially decreased when they also perceived themselves as accountable for the given recommendation. Our moderated mediation analysis indicated that the relation between advisor’s self-interest and the explicit strategic behavior (reduced transfer of conflicting information) can be explained by implicit strategic behavior (devaluation of conflicting information). But this was only the case when accountability was high – which confirm accountability again as moderator.

Unfortunately, findings of Study 2 do not exactly replicate findings of Study 1 (self-interest × type of information). One reason is that in participants with the concern of accountability higher responsibility was salient (attempt of manipulation), which weakened the effect regarding the experimental self-interest and participants did not differentiate between conflicting and supporting information as strong as in Study 1. However, we found convincing findings which showed that participants with high perceived accountability and without self-interest behave especially responsible for their clients regarding conflicting information – they increased transfer and evaluation of conflicting information. Thus, under high accountability without self-interest participant showed especially responsible for the client. But in combination with self-interest, the advisors acted in an even more self-interested way – they withhold and devalue information conflicting with their self-interest. These findings underline in our opinion the weakening effect of the self-interest manipulation.

## General Discussion

The present research examined the effect of incentives on two different forms of strategic behavior. Within two studies we could show that the promise to receive an incentive led to deception through explicit as well as implicit strategic behavior. The aim of Study 1 was to investigate the consequences of self-interest regarding information which are in conflict or in support with the self-interest. The results provided twofold evidence for *explicit strategic behavior*: firstly, self-interested advisors explicitly recommended the self-serving job option more often compared to those without specific interest. Secondly, we could observe that advisors passed on more supporting information and withhold more conflicting information from their clients compared to participants without self-interest. In Study 2, we measured beside self-interest also advisors’ perceived accountability. Our results indeed showed an interaction between self-interest and accountability regarding the transfer of conflicting information. In other words, we found that self-interested advisors increased their explicit strategic behavior by withholding conflicting information compared to advisors without self-interest when accountability was high. This was not the case when accountability was low.

Our findings regarding the explicit strategic behavior were in line with the described strategic behavior of PAT (Ross, 1973) which especially predicts “hidden information” as potential risk in relationships where information is distributed asymmetrically and the two parties have conflicting goals. Similar, Steinel and De Dreu’s (2004) findings showed that participants were less accurate when confronted with a competitive counterpart with opposed interests. However, our self-interested participants used withholding conflicting information and passing on supporting information as method to pursue self-interest and to bolster the self-interested decision. Similar in the study of Steinel and De Dreu (2004) this behavior could be observed to primarily handicap the other person and to enrich oneself. Our results indicate that advisors are motivated by the possibility to receive an incentive and therefore transfer information strategically and give strategic recommendation.

Furthermore, we provide evidence for *implicit strategic behavior*, which has so far not been investigated in past research. Thus, it is highly relevant to look at deception in its entirety – this means beside deception as explicit behavior also as bias in information processing. Referring to our results self-interested advisors were biased implicitly which again could be identified twofold: firstly, participants with self-interest evaluated information less in favor of clients’ needs compared to the control group. The interaction effect between self-interest and the information type (supporting vs. conflicting regarding self-interest) showed that advisors without self-interest wanted to find the best solution for the client and therefore enhanced evaluation of the conflicting information (supporting for the client) compared to the supporting information (conflicting for the client). This pattern disappeared in advisors with self-interest, who seemed not to take the perspective of the client and his needs into account. Secondly, self-interested advisors even remembered highly conflicting information worse than advisors without self-interest. Interestingly, the biased memory performance can also be explained by the evaluation of conflicting information. Among those participants who especially devalued the conflicting information in advance, high self-interest could significantly predict the bad memory regarding the conflicting information. This means Study 1 could provide first interesting evidence for implicit strategic behavior.

However, in Study 2 the interaction between self-interest and accountability was beside explicit strategic behavior also found in implicit strategic behavior. More specific, we stated in our analysis that self-interested advisors decreased the evaluation of conflicting information compared to advisors with low self-interest when accountability was high. There was no difference between high and low self-interest when accountability was low. Regarding memory performance of conflicting information also only high accountable participants showed a significant difference between high and low self-interest. Self-interested with high accountability could remember conflicting information worse. Taken together, our results supported the importance of implicit strategic behavior. Deception in advice-giving situation is also driven by biases in information processes like evaluating and remembering information which is even increased when accountability is high.

Our results regarding the implicit strategic behavior provide further support for Kunda’s (1990) assumption of biased information processing in favor of one’s wishes and desires – or in the current study to earn the incentive and pursue self-interest. So far, research provided evidence for self-interested participants to devalue arguments as less persuasive when its content was against their self-interest (Darke and Chaiken, 2005). We could additionally provide evidence that the self-interested information evaluation interacted with self-interest and led to worse memory performance regarding conflicting information. This means that especially those who devalued already conflicting information in advance remembered this information worse. In other words, these results suggested that bias in memory arises especially when information is not compatible with the self-interest and is evaluated therefore more negatively.

The findings of Study 2 additionally indicate the enhanced influence of self-interest on strategic behavior when accountability is high. So far past research showed under high accountability advisors’ search was more confirmation based and in line with preliminary decision (Jonas et al., 2005). And this research also identified impression motivation as directional goal why people are motivated to bias information. Our current results provided evidence that receiving an incentive function as a directional goal and led to a bias. The combination of high self-interest and high accountability enhanced this bias and led to a higher extant of strategic behavior. Highly accountable advisors seem to bias their information transfer in order to convince the client of the self-interested alternative.

Interestingly, in both studies we could confirm that implicit strategic behavior can predict to some extent explicit strategic behavior. Our mediation analysis indicated that the relation between advisor’s self-interest and the explicit strategic behavior (reduced transfer of conflicting information) can be explained by implicit strategic behavior (devaluation of conflicting information). In Study 2 this was also the case but only when accountability was high – which highlighted accountability as moderator again. Both mediation analyses are nice evidence that implicit actions explain partly the process of explicit strategic behavior. In other words, advisors might to some extent deceive themselves through biased information processing to justify their explicit strategic behavior afterward. The interesting findings of Shalvi et al. (2011) support this view: they found that the degree of lying depends on the extant of possible self-justification which participants emerge through biased information processing. This means in our study that especially the implicit strategy (the devaluation of conflicting information) justifies in turn the explicit strategic behavior (withholding of conflicting information). According to these results we must suppose that the promise of incentives can lead advisors to implicit strategic behavior which in turn leads to explicit strategic behavior. We have to take implicit actions more into account in order to understand explicit strategic behavior and deception. We will discuss especially the implications of this finding later.

Additionally to past research our results provide important evidence of the implicit strategic behavior which shows that advisors are influenced by their evaluation and memory. These are processes which advisors themselves can hardly control. Former research of such implicit processes also defined the term “directed forgetting” which especially explains reduced retrieval of unwanted memories or information (e.g., Freud, 1900/1964). However, this phenomenon should not lead to permanent damage of the information. Therefore, for future research it would be essential not only to test the recall of information but also if it can be recognized again (for overview: Baddeley et al., 2009). A further implicit phenomenon is the attention and which might be also directed through our motives. Isaacowitz (2006) exactly discuss this and describes attention as a tool of motivation. Eye-tracking studies provide some evidence that people are often strategic in their attentional preference and he assumed that “people guide their attention to information that can help them to achieve their goals and put away from stimuli that will not” (Isaacowitz, 2006, p. 68). Bias in attention might be also relevant in our study where especially self-interested participants could have used their attention as tool to guide their self-interested intention. For future research, especially eye-tracking studies can help us to understand how much attention self-interested participant pay to supporting vs. conflicting information and with how much effort they try to understand the match between the applicant and the different job alternatives.

With regard to theoretical implications, these findings identified a new aspect of advice-giving, because strategic behavior and deception was hardly discussed in advisor–client research (for overview: Bonaccio and Dalal, 2006). Although we know that clients accept and use advice of self-interested advisors to a lesser extent compared to advisors without specific interest (Jodlbauer and Jonas, 2011) and that besides advisor’s expertise and confidence also advisor’s good intention is highly relevant when evaluating the advisor’s recommendation quality (Bonaccio and Dalal, 2009). One exception is the research of Van Swol (2009) who manipulated two different motives – persuasion vs. quality – during the advice-giving process and could show that advisor’s motive to persuade manifested in using a high public confidence rating. They did that in a strategic way to convince the client because the private confidence rating differed significantly. Interestingly this attempt was successful in order to pursue clients. Our present research can confirm that advisors behave strategically. However, our results provide an extension of previous research and suggest that strategic behavior has an explicit and an implicit facet. Finally, we can state that the implicit strategic behavior is crucial because it can partly explain the explicit strategic behavior.

## Limitations

The reader should be aware that in Study 2 our manipulation for accountability did not work. Therefore, our simple slope analyses are also based on correlative data (including also self-interest, experimental self-interest was less convincing). As already discussed, one reason might be that in participants confronted with the manipulation higher responsibility was salient, which weakened the effect regarding the experimental self-interest and differentiation between conflicting and supporting information compared to Study 1. However, we could find convincing findings with simple slope analysis, which is a state-of-the-art analysis for moderation effects. Still, it is a limitation to use correlative data because there can be confounds which we are not aware of. Therefore, in future research it will be essential to manipulate accountability and at the same time self-interest successfully, so that results can be based on experimental manipulation.

A further limitation is that we used students and not real advisors. There is for example evidence, that real experts (physicians) search longer for an alternative explanation and could therefore reduce errors compared to novice (Krems and Zierer, 1994). However, there are also “costs of expertise”: Experts who decided for an alternative were more rigid and did not change their decisions easily (Sternberg, 1996). These findings indicate that especially for the practical implications it would be essential to test our hypothesis also with real advisors. Furthermore, regarding incentives it can be further essential to test an incentive that is common in this business and the real field of advisors.

## Practical Implications and Future Directions

In many advisor–client interactions incentives as explicit motivator are part of the business. Companies want to control the interests of the advisors and match them with their interests. For instance, even physicians, who are highly responsible for their clients, are in this situation. When physicians are rewarded with gifts or even get paid when supporting the interests of pharmaceutical companies (e.g., recommending a certain medication, referrals to clinical trials) they are at risk to behave strategically. This approach implies the risk that advisors clearly and explicitly subordinate the needs of the customer to their self-interest. Well, the explicit self-interested behavior aroused by incentives is known and in a way desired in this business sector of the pharmaceutical companies. Furthermore, for physicians this explicit strategic behavior seems maybe controllable and they feel not influenced in their objectivity. But based on our results self-interest is not limited to explicit and conscious acting. Moreover, the influence of incentives goes a step further and already influences their evaluation when searching and thinking about the best medication for their client and moreover they can later remember conflicting information regarding the medication worse. Our findings strongly indicate that advisors do not act independently of their more implicit processes of information processing. The implicit strategic behavior entail a high risk for clients and also for advisors’ themselves. It might be especially crucial how incentives are used. The promise of incentive connected with a certain alternative or product showed in our study evidence for deception. Based on the use of incentive within this study, strategic behavior might be especially high because of the connection between the incentive and a certain alternative (product engineer) or product, such as a certain medication. Further research in this field would be necessary to investigate different forms of providing incentives and how this lead to explicit, and implicit strategic behavior.

## Conclusion

In order to improve the understanding of deception our results indicated to take explicit and implicit strategic behavior into account. Advisors gave recommendation and transfer of information in self-interested strategic manner to deceive the client. The advisors also biased the information processing which can be seen as an implicit strategic way to deceive the client. Furthermore, the fact that the advisor should justify his/her recommendation even increased strategic behavior – explicit as well as implicit.

## Conflict of Interest Statement

The authors declare that the research was conducted in the absence of any commercial or financial relationships that could be construed as a potential conflict of interest.

## Footnotes

^1^Author of “Drive: The Surprising Truth About What Motivates Us.”

^2^The scale perceived self-interest of the manipulation check was used.

## Appendix

**Table A1:** **Information regarding the job alternatives differentiating between supporting and conflicting information regarding the client’s and the advisor’s interest**.

Information regarding…	Mechatronic engineer		Product engineer		Machinery engineer	
	Client	Advisor^a^	Client	Advisor	Client	Advisor
Job characteristics	+^b^	−^b^	−	−	+	−
Career opportunities	Neutral	Neutral	+	+	+	−
Further professional development	+	−	+	+	+	−
Salary	+	−	−	−	−	+
Competence needs	+	−	−	−	−	+
Labor-market situation	+	−	+	+	−	+

^a^For further analysis supporting and conflicting refer always to the advisor’s interest;

*^b^+ supporting, − conflicting*.

## References

[B1] Aiken L. S. West S. G. (1991). Multiple regression: Testing and interpreting interactions. Newbury Park: Sage

[B2] Baddeley A. Eysenck M. W. Anderson M. C. (2009). “Motivated forgetting,” in Memory, eds Baddeley A. Eysenck M. W. Anderson M. C. (New York: Psychology Press), 217–244

[B3] Boles T. L. Croson R. T. A. Murninghan J. K. (2000). Deception and retribution in repeated ultimatum bargaining. Organ. Behav. Hum. Decis. Process. 83, 235–259 10.1006/obhd.2000.2908 11056070

[B4] Bonaccio S. Dalal R. S. (2006). Advice taking and decision-making: an integrative literature review, and implications for the organizational sciences. Organ. Behav. Hum. Decis. Process. 101, 127–151 10.1016/j.obhdp.2006.07.001

[B5] Bonaccio S. Dalal S. D. (2009). Evaluating advisors: a policy-capturing study under conditions of complete and missing information. J. Behav. Decis. Mak. 23, 227–249 10.1002/bdm.649

[B6] Buller D. B. Burgoon J. K. (1994). “Deception: strategic and nonstrategic communication,” in Strategic Interpersonal Communication, eds Daly J. A. Wiemann J. M. (Hillsdale: Erlbaum), 191–223

[B7] Buller D. B. Burgoon J. K. (1996). Interpersonal deception theory. Commun. Theory 6, 203–242 10.1111/j.1468-2885.1996.tb00127.x

[B8] Burgoon J. K. Buller D. B. Ebesu A. Rockwell P. White C. (1996). Testing interpersonal deception theory: effects of suspicion on nonverbal behavior and relational messages. Commun. Theory 6, 243–267 10.1111/j.1468-2885.1996.tb00132.x

[B9] Darke P. R. Chaiken S. (2005). The pursuit of self-interest: self-interest bias in attitude judgment and persuasion. J. Pers. Soc. Psychol. 89, 864–883 10.1037/0022-3514.89.6.864 16393021

[B10] De Cremer D. Van Dijk E. (2009). Paying for sanctions in social dilemmas: the effects of asymmetry of endowments and accountability. Organ. Behav. Hum. Decis. Process. 109, 45–55 10.1016/j.obhdp.2009.01.004

[B11] Ditto P. H. Scepansky J. A. Munro G. D. Apanovitch A. M. Lockhart L. K. (1998). Motivated sensitivity to preference-inconsistent information. J. Pers. Soc. Psychol. 75, 53–69 10.1037/0022-3514.75.1.53

[B12] Dunning D. (1999). A new look: motivated social cognition and the schematic representation of social concepts. Psychol. Inq. 10, 1–11 10.1207/s15327965pli1001_15

[B13] Eisenhardt K. (1989). Agency theory: an assessment and review. Acad. Manage. Rev. 14, 57–74 10.5465/AMR.1989.4279003

[B14] Freud S. (1900/1964). The Interpretation of Dreams. New York: Basic Books

[B15] Isaacowitz D. M. (2006). Motivated gaze: the view from the gazer. Curr. Dir. Psychol. Sci. 15, 68–72 10.1111/j.0963-7214.2006.00409.x

[B16] Jodlbauer B. Jonas E. (2011). Forecasting clients’ reactions: how does the perception of strategic behavior influence the acceptance of advice? Int. J. Forecast. 27, 121–133 10.1016/j.ijforecast.2010.05.008

[B17] Johnson B. T. Eagly A. H. (1989). The effects of involvement on persuasion: a meta-analysis. Psychol. Bull. 106, 290–314 10.1037/0033-2909.106.2.290

[B18] Jonas E. Frey D. (2003). Information search and presentation in advisor–client interactions. Organ. Behav. Hum. Decis. Process. 91, 154–168 10.1016/S0749-5978(03)00059-1

[B19] Jonas E. Schulz-Hardt S. Frey D. (2005). Giving advice or making decisions in someone else’s place – the influence of impression, defense and accuracy motivation on the search for new information. Pers. Soc. Psychol. Bull. 31, 977–990 10.1177/0146167204274095 15951368

[B20] Jonas E. Traut-Mattausch E. Frey D. Greenberg J. (2008). The path or the goal? Decision vs. information focus in biased information seeking after preliminary decisions. J. Exp. Soc. Psychol. 44, 1180–1186 10.1016/j.jesp.2008.02.009

[B21] Jungermann H. (1999). “Advice-giving and taking,” in Proceedings of the 32nd Hawaii International Conference on System Sciences (HICSS-32). [CD-ROM] (Maui: Institute of Electrical and Electronics Engineers, Inc.). Available at: http://www.gp.tu-berlin.de/Users/j/jungermann/Publications/Advice%20giving.html

[B22] Krems F. Zierer C. (1994). Sind Experten gegen kognitive Täuschungen gefeit? Zur Abhängigkeit des confirmation bias von Fachwissen. Z. Exp. Angew. Psychol. 41, 98–115 8178540

[B23] Kruglanski A. W. (1989). Lay epistiemics and human knowledge: Cognitive and motivational bases. New York: Plenum Press

[B24] Kruglanski A. W. Bélanger J. J. Chen X. Köpetz C. Pierro A. Manetti L. (2012). The energetic of motivated cognition: a force-field analysis. Psychol. Rev. 119, 1–20 10.1037/a0025488 21967165

[B25] Kunda Z. (1987). Motivated inference: self-serving generation and evaluation of causal theories. J. Pers. Soc. Psychol. 53, 636–647 10.1037/0022-3514.53.4.636

[B26] Kunda Z. (1990). The case for motivated reasoning. Psychol. Bull. 108, 480–498 10.1037/0033-2909.108.3.480 2270237

[B27] Kunda Z. Sanitioso R. (1989). Motivated change in the self-concept. J. Exp. Soc. Psychol. 25, 272–285 10.1016/0022-1031(89)90023-1

[B28] Lerner J. S. Tetlock P. E. (1999). Accounting for the effects of accountability. Psychol. Bull. 125, 255–275 10.1037/0033-2909.125.2.255 10087938

[B29] McDonald H. E. Hirt E. R. (1997). When expectancy meets desire: motivational effects in reconstructive memory. J. Pers. Soc. Psychol. 72, 5–23 10.1037/0022-3514.72.1.5 9008371

[B30] Petty R. E. Cacioppo J. T. (1990). Involvement and persuasion: tradition versus integration. Psychol. Bull. 107, 367–374 10.1037/0033-2909.107.3.367

[B31] Preacher K. J. Rucker D. D. Hayes A. F. (2007). Addressing moderated mediation hypotheses: theory, methods, and prescriptions. Multivar. Behav. Res. 42, 185–227 10.1080/00273170701341316 26821081

[B32] Ross S. A. (1973). The economic theory of agency: the principal’s problem. Am. Econ. Rev. 62, 134–139

[B33] Sanitioso R. Kunda Z. Fong G. T. (1990). Motivated recruitment of autobiographical memories. J. Pers. Soc. Psychol. 59, 229–241 10.1037/0022-3514.59.2.229 2213492

[B34] Shalvi S. Dana J. Handgraaf M. J. J. De Dreu C. K. W. (2011). Justified ethicality: observing desired counterfactuals modifies ethical perceptions and behavior. Organ. Behav. Hum. Decis. Process. 115, 181–190 10.1016/j.obhdp.2011.02.001

[B35] Simonson I. Staw B. M. (1992). Deescalation strategies: a comparison of techniques for reducing commitment to loosing courses of action. J. Appl. Psychol. 77, 419–426 10.1037/0021-9010.77.4.419

[B36] Steinel W. De Dreu C. K. W. (2004). Social motives and strategic misrepresentation in social decision making. J. Pers. Soc. Psychol. 86, 419–434 10.1037/0022-3514.86.3.419 15008646

[B37] Sternberg R. J. (1996). “Costs of expertise,” in The Road to Excellence, ed. Ericsson K. A. (Mahwah: Lawrence Erlbaum Associates), 347–354

[B38] Stone D. N. Ziebart D. A. (1995). A model of financial incentive effects in decision making. Organ. Behav. Hum. Decis. Process. 61, 250–261 10.1006/obhd.1995.1020

[B39] Taber C. S. Lodge M. (2006). Motivated skepticism in the evaluation of political beliefs. Am. J. Pol. Sci. 50, 755–769 10.1111/j.1540-5907.2006.00214.x

[B40] Van Swol L. M. (2009). The effects of confidence and advisor motives on advice utilization. Communic. Res. 36, 857–873 10.1177/0093650209346803

